# Associations between Objective and Subjective Housing Status with Individual Mental Health in Guangzhou, China

**DOI:** 10.3390/ijerph18030930

**Published:** 2021-01-21

**Authors:** Lijian Xie, Suhong Zhou, Lin Zhang

**Affiliations:** 1 School of Tourism and Geography Science, Qingdao University, Qingdao 266071, China; xielijian@qdu.edu.cn; 2 Research Center for Marine Management Strategy, Qingdao University, Qingdao 266071, China; 3 School of Geography and Planning, Sun Yat-sen University, Guangzhou 510275, China; eeszsh@mail.sysu.edu.cn; 4 Guangdong Provincial Engineering Research Center for Public Security and Disaster, Guangzhou 510275, China; 5 Institute of Studies of Greater Bay Area, Guangdong University of Foreign Studies, Guangzhou 510006, China

**Keywords:** objective housing status, subjective housing status, mental health, moderating effects

## Abstract

Housing is an important social determinant of mental health. However, few studies simultaneously measure the objective housing status (i.e., housing tenure, living space, housing conditions, and housing stability) and subjective housing status (i.e., housing satisfaction) as well as examine their effects on people’s mental health (i.e., stress, anxiety, and depression). Thus, using a sample size of 1003 participants by two-stage random sampling survey in Guangzhou, China, this study applies multivariate ordinary least square regression models to comprehensively explore and compare the associations between objective and subjective housing status with mental health, and then analyze the moderating effects of subjective housing status on the relationships between objective housing status and mental health. The findings suggest that there are significant differences in people’s mental health based on different housing status. The subjective housing status can better explain the variances in mental health than objective housing status. Also, subjective housing status may partly mitigate the adverse impacts of objective housing disadvantages on some aspects of an individual’s mental health. Therefore, housing improvement policies and public health initiatives should be designed based on a comprehensive account of objective and subjective housing characteristics as well as their influences on specific aspects of mental health.

## 1. Introduction

### 1.1. Background

China’s urbanization over the last decades has been unprecedented in human history. Approximately 260 million people have moved from the countryside to the city, which greatly promotes rapid economic growth and development [1]. China’s urbanization rate has from 17.92% in 1978 to 60.6% in 2019. This unprecedented urbanization wave has not only created a miracle of economic growth in recent decades, but it has also brought a series of challenges and problems, especially in China’s megacities. Housing is one of the key problems with rapid urbanization. With the deepening of China’s urbanization, housing problems are becoming more serious and complicated, such as high housing prices, overcrowded housing, residential segregation, job-housing imbalance, and housing inequality [2]. In terms of the housing price problem that urban residents are generally more concerned about, a study of housing prices in major Chinese cities has found that for every 1% increase in the level of urbanization, the housing prices are driven up by 0.343% [3], which will induce housing affordability stress that may affect people’s mental health.

Housing has long been understood as a social determinant of mental health in social epidemiological studies [4,5], but the majority of prior studies have mainly involved the effects of one dimension of housing status (i.e., objective or subjective housing status) on mental health, therefore, there is still very limited research to simultaneously examine the health impacts of both objective and subjective housing status. We recognize that knowledge about the impacts of both objective and subjective housing status on individual mental health is necessary for addressing housing-related problems, and using only one dimension of housing status may engender a misleading and incomplete picture of health determinants. More importantly, pathways through which housing-related objective inadequacies such as poor conditions affect mental health are not likely to be the same as those for a housing-related subjective status such as housing satisfaction that may influence people’s mental health [6]. As for mental health, many previous studies have investigated the effects of housing status on self-rated general mental health. According to past research [7,8,9], individuals’ mental health can be represented by different aspects such as stress, anxiety, and depression. Yet, mixing these aspects or only using an aggregated index/score (i.e., self-rated general mental health) may not be conducive to clarifying the impacts of housing status on mental health. Therefore, to fully reflect the impacts of housing status on an individual’s mental health, it is necessary to comprehensively examine the effects of objective and subjective housing status on multiple aspects of mental health (i.e., stress, anxiety, and depression). Additionally, the moderating effects of subjective housing conditions on the relationships between objective housing conditions and mental health may differ when different aspects of mental health are considered. However, to the best of our knowledge, these have not been adequately studied so far.

### 1.2. Literature Review

We know that individuals spend a substantial amount of time at their homes [10,11]. A growing body of evidence has established that inadequate housing and poor conditions are correlated to worsening mental health [12,13]. However, the potential path linking housing status to individual health is quite complex [14]. Many aspects of housing status have an impact on mental health, but scholars have paid more attention to the variables of objective housing status: housing tenure or ownership, housing conditions or quality, housing size or living space, and housing stability or residential mobility.

Existing studies on the associations between housing tenure and mental health mainly focus on the differences in mental health outcomes between homeowners and tenants. Extensive evidence of these relationships has emphasized the social benefits of housing ownership where homeowners have better mental health outcomes than tenants [15,16]. Some studies in the UK indicate that housing tenure can explain differences in mental health (i.e., anxiety, depression) after controlling for socio-demographic variables [16,17,18]. However, other studies claim that there is no intrinsic relationship between homeowners and tenants in psychological well-being [19]. These inconsistent conclusions may suggest that it is worthwhile to further explore the differences in the mental health impacts of housing tenure among different groups.

The negative effects that poor housing conditions have on people’s mental health outcomes are well documented in prior literature [20,21,22]. In detail, poor housing conditions (e.g., lack of basic living facilities; damp, cold, and moldy housing) are associated with mental illness (e.g., anxiety, depression) [23,24]. Although the existing research has relatively consistent conclusions, few scholars suggest that most existing studies only use poor housing condition indexes with a very limited number of measurements, which may not accurately estimate the influences of housing conditions on an individual’s health [25].

Similarly, inadequate living space or overcrowded housing also harms individuals’ mental health, which has been widely reported [26,27]. Overcrowded housing is found to be independently related to self-assessed health and psychological distress [28,29]. Two paths for the impacts of housing size/living space on mental health have been proposed: the first one is that housing size affects mental health through the facilitation of activities and values, and the second one is that housing size enhances mental health by improving people’s social status [30].

Previous research on housing instability mainly focuses on the effects of residential mobility on mental health in adolescence, while the studies on adults are relatively fewer [31]. Available evidence generally shows that adolescents who have experienced housing changes in their early life are more likely to have mental ill-health [32]. Additionally, some studies indicate that housing stability, captured by the months of living in the current property, is significantly negatively correlated with adults’ mental illness [33].

To the best of our knowledge, only several studies have explored the associations between people’s housing satisfaction and their mental health, and their results are mixed. For instance, a longitudinal study in Germany indicates that the change in housing satisfaction is positive, yet rather weakly associated with the change in health [34]. In contrast, a study examines the association between housing satisfaction and mental health and finds that the pathway of housing satisfaction is not significantly linked with mental health in structural equation modeling [35]. Similarly, a cross-sectional study focusing on multidimensional housing satisfaction indicates that housing satisfaction, overall, is not to be predictive of subjective psychological distress (e.g., psychoticism, depression, and anxiety) [36].

**Figure 1:**
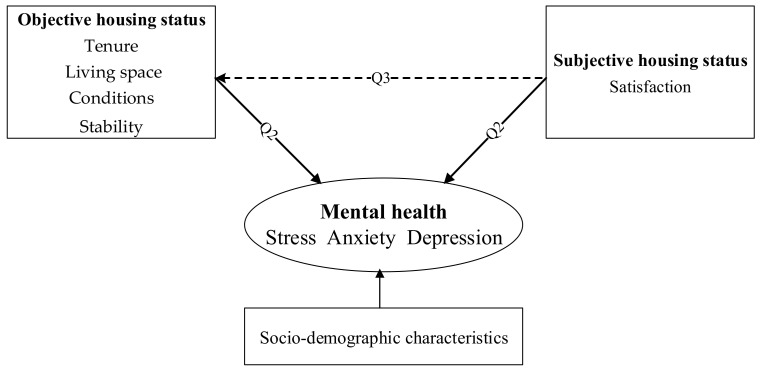
Conceptual framework.

Considering the public health consequences of housing disadvantages that emerge from rapid urbanization in China as well as the gaps identified in existing literature, the present study proposes a conceptual framework (Figure 1) and aims to address the following questions:

Question 1: Are there differences in the mental health of urban residents living in a typical megacity in China, based on different aspects of housing status?

Question 2: How does objective and subjective housing status respectively affect people’s mental health? Is there any difference in the explained variance of objective and subjective housing status on different aspects of mental health (i.e., stress, anxiety, and depression)?

Question 3: Does subjective housing status exert the moderating effects on the relationships between objective housing status and mental health?

## 2. Data and Methods

### 2.1. Study Area

**Figure 2:**
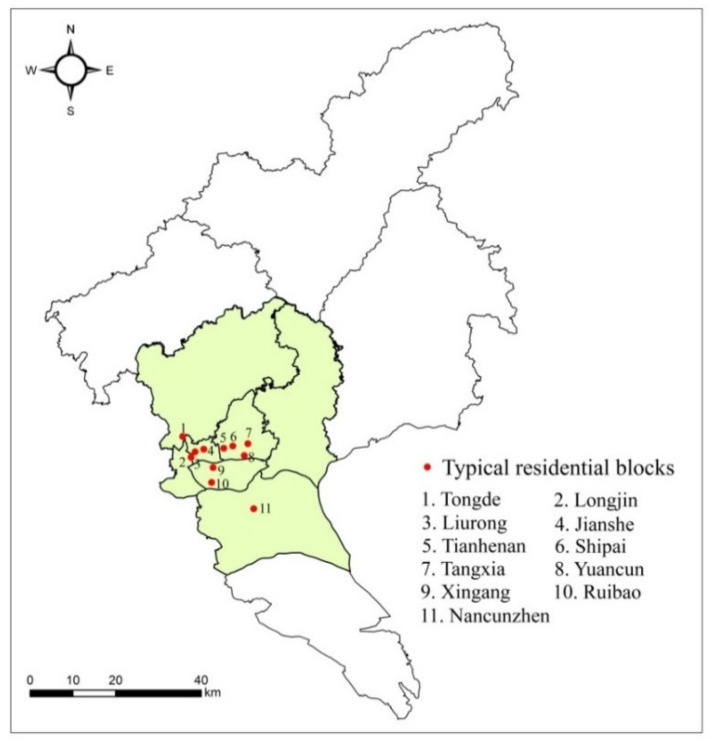
Study area and typical residential blocks.

Guangzhou, one of the most important megacities in China, is the capital of Guangdong Province. The city has a total area of 7434.4 km^2^ and a permanent population of about 14.49 million in 2017 (http://www.gz.gov.cn/zwfw/zxfw/gysy/content/mpost_2859028.html). After China’s reform and opening-up in 1978, Guangzhou has experienced rapid urbanization and housing reform and formed a rich structural landscape of housing types (e.g., historical block, affordable housing, *danwei* compounds [which refer to a typical form of residential organization in the period of China’s planned economy], commercial housing, and informal housing community). Thus, this paper chose Guangzhou as the representative study area, in which 11 residential blocks were investigated. These residential blocks were located in the central, transitional, and marginal areas of the city, including Liwan, Yuexiu, Haizhu, Tianhe, Baiyun, Huangpu, and Panyu districts (Figure 2).

### 2.2. Data

This study was performed using data from a questionnaire of residents in Guangzhou, China. The questionnaire was conducted by a professional survey agency and lasted from March to August 2017. To be specific, according to the size of permanent residents in each block reported in the Sixth National Census of China, its location, history, and housing types, 11 residential blocks were selected first, from which participants were randomly selected. When designing the questionnaire, we chose some reliable and validated scales or items which have been commonly used in previous surveys or studies. A detailed structured questionnaire was administered by trained interviewers from the professional survey agency, and it collected information from participants including socio-demographic characteristics, housing status, and self-rated mental health, etc. All participants provided informed consent. The detailed selection process and questionnaire have been described elsewhere [37]. After checking the original data, questionnaires with incomplete and inconsistent responses were excluded from the study. The response rate of the questionnaire was about 75%. Finally, this study included 1003 adults who have completed information on socio-demographic characteristics, objective and subjective housing status, and mental health-related indicators.

#### 2.2.1. Covariates: Socio-Demographic Characteristics

This paper selects the following covariates: age, gender (male, female), education level (primary school or below, junior high school, senior high school, bachelor degree, master degree or above), personal monthly income (Yuan) (≤2999, 3000–4999, 5000–8999, 9000–11,999, ≥12,000), and marital status (married, single [unmarried, divorced, widowed]), and *hukou* [which refers to China’s household registration system] (local *hukou*, non-local *hukou*). 

The average age of respondents was around 36 years (S.D. = 9.65). There were 501 (50.0%) males and 502 (50.0%) females. 66.1% of respondents had a bachelor’s degree or above. The personal monthly income of most respondents was less than 9000 Yuan. Additionally, 80.1% of respondents were married, and 75.4% of respondents had local *hukou*.

#### 2.2.2. Independent Variables: Objective and Subjective Housing Status

Drawing upon previous literature [38,39,40], objective housing status included housing tenure, living space, housing conditions, and housing stability. Subjective housing status mainly included housing satisfaction. Specifically, housing tenure was measured with a dichotomous variable differentiating between tenant and homeowner. The living space was determined by dividing the total area of each housing unit by the number of inhabitants of each corresponding household. Participants whose living space was less than 20 m^2^ were regarded as living in overcrowded housing. Housing conditions were assessed by the question “Does your accommodation have any of the following facilities (access to running water, separate washroom, shower, separate kitchen, air conditioning/heating equipment)?” These facilities were all categorized as “available” or “unavailable”. If a participant answered “unavailable” to any of the five facilities, it was considered as living with poor housing conditions [41,42]. Housing stability level was captured by the question “Have you changed your place of residence in the past 5 years?” Participants who answered “Yes” were defined as living in unstable housing. Additionally, housing satisfaction was assessed by the question “How satisfied are you with your current dwelling?” with responses provided on a 5-point Likert scale (very unsatisfied, unsatisfied, general, satisfied, very satisfied). This distinguished between “unsatisfied housing” (responses of very unsatisfied or unsatisfied) and “satisfied housing” (responses of general, satisfied, or very satisfied). All variables of housing status were mean-centered in multivariate analysis to avoid multicollinearity problems as much as possible.

#### 2.2.3. Outcome Variables: Mental Health

According to prior research [7,8,9], mental health variables included stress, anxiety, and depression in this paper. For stress, participants were asked “How often do you feel stressed during the last year?” and could answer from 5 options (always, often, sometimes, seldom, never). Participants who responded with “always” or “often” were distinguished as having frequently-perceived stress. Anxious and depressive symptoms were assessed via the Symptom Check List-90 (SCL-90) [43,44], a self-reported scale with each item rated on a 5-point Likert scale (from 1 “not at all” to 5 “extremely serious”). The SCL-90 scale comprised a 10-item subscale for anxiety (SCL-90-A) and a 13-item subscale for depression (SCL-90-D). Cronbach’s alpha of the two subscales in this study was 0.908 and 0.921, respectively. For anxiety and depression, the subscale average scores ≥2 commonly indicated a potentially high level of psychological symptoms [45]. An SCL-90-A average score ≥2 was defined as having anxious symptoms, and an SCL-90-D average score ≥2 was defined as having depressive symptoms.

The descriptive statistics of variables used to examine the associations between objective and subjective housing status with individual mental health are shown in Table 1.

### 2.3. Statistical Analyses

This paper first used percentages and frequency counts to summarize the participants’ socio-demographic characteristics, housing status, and mental health. Second, the Pearson χ^2^ test was applied to examine the differences in participants’ mental health by their housing status (housing tenure, living space, housing conditions, housing stability, and housing satisfaction). Third, multivariate ordinary least square (OLS) regression models were used to explore the impacts of objective housing status (i.e., housing tenure, living space, housing conditions, and housing stability) and subjective housing status (i.e., housing satisfaction) on stress, anxiety, and depression, respectively. Finally, in order to examine the moderating effects of subjective housing status on the relationships between objective housing status and individual mental health, the interaction terms of housing satisfaction and housing tenure, living space, housing conditions, and housing stability were added to the corresponding multivariate OLS regression models. The regression coefficients and standard errors were calculated after controlling for socio-demographic characteristics.

## 3. Results

### 3.1. Differences in Mental Health

This paper analyzed the differences in individuals’ mental health based on their housing status (Table 2). People who had a different status of housing tenure (tenant or homeowner) and living space (overcrowded housing or non-overcrowded housing) were more likely to have significantly different mental health, that is, in aspects of stress, anxiety, and depression. Comparing residents with unstable housing and those with stable ones, they had considerable differences in anxiety symptoms. Moreover, people who were satisfied with their housing tended to have a significantly different level of perceived stress when compared with those who were not satisfied with housing.

### 3.2. Associations between Objective and Subjective Housing Status with Mental Health

This study separately analyzed and compared the impacts of objective and subjective housing status on people’s mental health (i.e., stress, anxiety, and depression), and then explored whether there were differences in the explained variance of objective and subjective housing status on different aspects of mental health (i.e., stress, anxiety, and depression). Table 3, Table 4 and Table 5 show the results of multivariate OLS regression analysis for stress, anxiety, and depression, respectively, after controlling for socio-demographic characteristics.

#### 3.2.1. Associations between Housing Status and Three Aspects of Mental Health

Concerning stress (Table 3), Model 1 showed that housing tenure was negatively related to people’s perceived stress, which indicated that tenants reported less stress than homeowners. Additionally, Model 2 showed that the living space was negatively correlated with people’s perceived stress. It suggested that for every 1 m^2^ increase in people’s average living space, their perceived stress decreased by 0.016 units. Similarly, there was a negative relationship between housing satisfaction and stress in Model 5. This indicated that people reported less perceived stress as housing satisfaction increased. As for anxiety (Table 4) and depression (Table 5), multivariate OLS regression analyses showed similar results. Specifically, living space (Model 7 and Model 12) and housing satisfaction (Model 10 and Model 15) were significantly and negatively correlated with anxiety and depression, respectively. These results demonstrated that people tend to perceive lower levels of anxiety and depression with improvements in living space and housing satisfaction. In Model 9 and Model 14, there were significant negative relationships between housing stability with anxiety and depression, respectively. This meant that people who lived in unstable housing had higher levels of anxiety and depression than people who lived in stable housing.

#### 3.2.2. Explained Variance of Objective and Subjective Housing Status on Mental Health

Table 3, Table 4 and Table 5 also show the *R*^2^ in regression estimation of objective and subjective housing status on individuals’ mental health. The variances in stress explained by objective housing status (i.e., housing tenure, living space, housing conditions, and housing stability) and subjective housing status (i.e., housing satisfaction) were 2.1%, 2.8%, 1.3%, 1.3%, and 2.9%, respectively (Table 3). The variances in anxiety explained by objective and subjective housing status were 5.5%, 6.1%, 5.6%, 5.8%, and 6.3%, respectively (Table 4). In addition, 5.8%, 6.1%, 6.0%, 6.2%, and 6.3% of the variances in depression were explained by objective and subjective housing status, respectively (Table 5). In summary, the explained variance of subjective housing status on mental health was slightly higher than that of objective housing status. These results indicated that subjective housing status may be a better predictor of individual mental health than objective housing status.

### 3.3. The Moderating Effects of Subjective Housing Status

This research also analyzed the moderating effects of housing satisfaction as subjective housing status on the relationships between objective housing status and three aspects of mental health (i.e., stress, anxiety, and depression). Table 6, Table 7 and Table 8 illustrate the results of multivariate OLS regression models with a moderating variable (i.e., housing satisfaction) and interaction terms (i.e., housing tenure * housing satisfaction, living space * housing satisfaction, housing conditions * housing satisfaction, and housing stability * housing satisfaction), after controlling for socio-demographic characteristics. The R^2^ values of some regression models in Table 6, Table 7 and Table 8 were greater than their respective corresponding models (Taking stress as an example, the R^2^ increased from 0.055 (in Model 16 of Table 6) to 0.058 (in Model 17 of Table 6)), which indicated that these models were more robust and the explained variance of objective housing status on mental health was stronger after adding the moderating variable and interaction term. 

As for stress (Table 6), Model 17 showed that the main effects of housing tenure and satisfaction remained significant after adding the interaction term of housing tenure and housing satisfaction. Also, the interaction term (housing tenure * housing satisfaction) was positive and significant. These results demonstrated that with an increase in housing satisfaction, the perceived stress of tenants decreased considerably faster than that of homeowners, that is, as housing satisfaction improved, tenants benefitted more than homeowners. In other words, this result indicated that housing satisfaction may moderate the impacts of housing tenure on people’s stress.

Concerning anxiety and depression, Model 25 in Table 7 and Model 30 in Table 8 showed that the main effects of housing stability and housing satisfaction remained after the addition of interaction term (housing stability * housing satisfaction). The interaction term was also positive and significant. These results showed that with the improvement of housing satisfaction, the decline in levels of anxiety and depression was considerably faster for people who lived in unstable housing than for those who lived in stable housing, that is, as housing satisfaction improved, dwellers living in unstable housing benefitted more than those living in stable housing. It suggested that although people who lived in unstable housing may have anxious and depressive symptoms, their anxiety and depression could be reduced when they were satisfied with their housing. In other words, housing satisfaction may reduce the negative effects of poor housing stability on individuals’ anxiety and depression.

Model 23 in Table 7 and Model 28 in Table 8 also demonstrated that the main effects of living space and housing satisfaction were still significant after adding the interaction term of living space and housing satisfaction. Moreover, the interaction term (living space * housing satisfaction) was negative and significant. These results showed that the effects of an increase in living space and improved housing satisfaction on anxiety and depression were mutually compensating. It suggested that people who lived in overcrowded housing may reduce perceived anxiety and depression when they were satisfied with their dwellings, namely, housing satisfaction may mitigate the negative impacts of overcrowded housing on people’s anxiety and depression.

In general, subjective housing status (i.e., housing satisfaction) exerted significant moderating effects on the associations between some objective housing variables (i.g., housing tenure, living space, and housing stability) and some aspects of individuals’ mental health. In other words, housing satisfaction may partly mitigate the adverse impacts of objective housing disadvantages on some aspects of people’s mental health.

## 4. Discussion

This study aims at advancing the literature on housing status and different aspects of mental health by expanding measurement dimensions of housing status (i.e., objective and subjective housing status) and exploring the adverse health effects of housing disadvantages for adults in urban China. Housing disadvantages in terms of housing tenure and conditions may not lead to poor mental health. However, housing disadvantages in terms of living space, stability, and satisfaction are more likely to affect mental health. The research findings indicate that objective and subjective housing status may influence three aspects of mental health (i.e., stress, anxiety, and depression), respectively. More importantly, the subjective housing status can better explain the variances in mental health than objective housing status. Additionally, the subjective housing status plays a moderating role in the relationships between objective housing status and individuals’ mental health.

It is interesting to note that tenants are more likely to have less perceived stress than homeowners, which is inconsistent with existing literature. Prior studies in developed countries typically indicate that housing tenure significantly affects mental health [46,47], and generally highlights that homeowners have better mental health than tenants [48,49]. It may be due to the influences of traditional Chinese culture, which emphasizes the concept of home, that is, to own a house means to have a home. Thus, some people are forced to spend a large part of their income on buying houses, which may lead to economic pressure and further affects their health status. In other words, housing affordability stress is a risk factor for an individual’s mental health [50,51,52].

Consistent with the conclusions of many previous studies [9,53], this paper also finds significant correlations between living space and mental health, respectively. Dwellers who live in overcrowded housing are found to have a higher risk of frequently-perceived stress, anxiety, and depression than those who live in non-overcrowded housing. As we all know, adequate living space includes being able to have enough privacy [22,54]. However, living in overcrowded environments can lead to excessive social stimulation, which in turn causes withdrawal responses accompanied by feelings of less perceived support, less perceived control, less social affection [55], helplessness, hopelessness, and sadness [56] that culminate in anxiety and depression. Additionally, life sciences have indicated that stress is the intermediary agent between urban life (e.g., overcrowding in housing) and mental illness [33].

To our surprise, this study does not find a significant statistical relationship between housing conditions and three aspects of mental health (i.e., stress, anxiety, and depression), which is inconsistent with most existing analyses. Many studies demonstrate that poor housing conditions are inimical to mental health [57,58]. One possible explanation for our finding is that these housing facilities (e.g., access to running water, separate washroom, shower, separate kitchen, air conditioning/heating equipment) have been widely available in urban housing in China, so that in this study, most respondents have good housing conditions, and thus their mental health level may not be affected by the improvement of housing conditions. Another possible explanation is that some unobserved factors may suppress or mitigate the impacts of poor housing conditions on individuals’ mental health, such as neighborhood cohesion, neighborhood satisfaction [59], social capital [60,61], etc.

Housing stability has a significantly positive impact on mental health, which means that people with unstable housing are more likely to have anxious and depressive symptoms. This finding is similar to what has been argued elsewhere [62]. A possible interpretation for the results is that people at different periods of their life may have different motives or reasons for residential mobility, in other words, whether they move voluntarily or passively. In this paper, young dwellers are more likely to move frequently (i.e., the regression coefficient of age and housing stability is 0.008, *p* < 0.001), since their purposes of moving may be to passively seek a corresponding education for their children or to change a job due to the economic pressure.

There are significant and positive associations between housing satisfaction and mental health (i.e., stress, anxiety, and depression), which are consistent with other research by Knöchelmann and colleagues [34]. The relationships between housing satisfaction and health outcomes may be explained by the idea of a psychosocial pathway, for instance, the feelings of housing satisfaction caused by social comparisons can contribute to the development of a sense of identity and attachment, and strengthen the feelings of safety and control, which in turn lead to healthy habits and psychological benefits [54].

In total, the objective and subjective housing status have different explained variance on stress, anxiety, and depression respectively. Additionally, the subjective housing status (e.g., housing satisfaction) partly alleviates the negative impacts of poor objective housing status on people’s mental health. It may inform that the effects of objective and subjective housing status and their interactions on mental health should be considered comprehensively when formulating policies aimed at improving housing status and promoting people’s mental health level.

This study makes meaningful theoretical contributions to the limited body of literature exploring objective and subjective housing status in relation to mental health. First of all, this research further deepens the understanding of the relationships between housing status and mental health through comprehensively exploring the impacts of subjective and objective housing status on different aspects of mental health (i.e., stress, anxiety, and depression). Secondly, this study finds that different dimensions of housing status have different impacts on different aspects of individuals’ mental health, and subjective housing status plays a significant moderating role in the relationship between objective housing status and individuals’ mental health.

Despite the significance of the methodological approaches and findings, this paper also has several limitations. First, this is a cross-sectional study, meaning that the cause cannot be inferred from the results. More longitudinal studies are required to explore the causal relationship between housing status and an individual’s health. Second, the featured population of this study is generally characterized by a better socio-demographic and housing scene than the average from the census, which limits its generalizability. Thus, more studies involving significant population heterogeneity and cultural affinities are required to validate these findings. Third, due to the lack of differences in the existing housing condition indicators among groups, future research needs to develop new indicators to accurately grasp the nature of housing conditions in urban China. Fourth, in understanding the relationship between housing instability and mental health, future research should further distinguish the intrinsic motivation of housing mobility to analyze the underlying mechanism of housing mobility affecting mental health.

## 5. Conclusions

The evidence from this study reinforces that housing is a key social determinant of mental health. There are significant differences in people’s aspects of mental health based on different attributes of their housing status. Exposure to housing disadvantages in living space, housing stability, and housing satisfaction are significantly associated with poor mental health (i.e., stress, anxiety, and depression), but there is no similar conclusion in terms of housing tenure and housing conditions. Compared with objective housing status, subjective housing status is a better predictor of an individual’s mental health. Also, subjective housing status moderates the relationships between objective housing status and mental health. Namely, subjective housing status (housing satisfaction) may partly mitigate the adverse effects of objective housing disadvantages on some aspects of mental health. These research findings can be used to optimize existing regulations and bring more attention to the specific dimension of housing status which has important impacts on public health in policy-making processes. As some scholars have argued, “Where we live represents a range of individual and local level attributes, which needs concern for compositional and contextual place-effects on health” [17].

## Figures and Tables

**Table 1:** Descriptive statistics of all variables *(n* = 1003).

Variables		*n*	Mean/%
Socio-Demographic Characteristics			
Age		1003	36.4 (9.65)
Gender	Male	501	50.0%
	Female	502	50.0%
Education level	Primary school or below	1	0.1%
	Junior high school	63	6.3%
	Senior high school	276	27.5%
	Bachelor degree	654	65.2%
Personal monthly income (Yuan)	≤2999	12	1.2%
	3000–4999	322	32.1%
	5000–8999	487	48.5%
	9000–11,999	75	7.5%
	≥12,000	107	10.7%
Marital status	Married	803	80.1%
	Single	200	19.9%
Hukou	Local *hukou*	756	75.4%
	Non-local *hukou*	247	24.6%
**Mental Health**			
Stress		1003	2.5
Anxiety		1003	17.0
Depression		1003	21.1
**Housing Status**			
Housing tenure	Tenant	131	13.1%
	Homeowner	872	86.9%
Living space (m^2^)		1003	25.0 (7.73)
Housing conditions	Poor housing conditions	9	0.9%
	Good housing conditions	994	99.1%
Housing stability	Unstable housing	241	24.0%
	Stable housing	762	76.0%
Housing satisfaction		1003	4.0

**Table 2:** Differences in individual mental health outcomes by housing status.

Variables	Stress			Anxiety			Depression		
	Frequently-Perceived Stress (*n =* 165)	No Frequently-Perceived Stress (*n* = 838)	*p*	Having Anxious Symptoms (*n* = 242)	No Anxious Symptoms (*n* = 761)	*p*	Having Depressive Symptoms (*n* = 207)	No Depressive Symptoms (*n* = 796)	*p*
**Housing tenure**									
Tenant	18.3%	81.7%	0.040	32.8%	67.2%	0.013	31.3%	68.7%	0.001
Homeowner	16.2%	83.8%		22.8%	77.2%		19.0%	81.0%	
**Living space**									
Overcrowded housing	16.1%	83.9%	0.000	29.2%	70.8%	0.021	25.9%	74.1%	0.011
Non-overcrowded housing	16.6%	83.4%		22.2%	77.8%		18.7%	81.3%	
**Housing conditions**									
Poor housing conditions	11.1%	88.9%	0.932	66.7%	33.3%	0.130	77.8%	22.2%	0.101
Good housing conditions	16.5%	83.5%		23.7%	76.3%		20.1%	79.9%	
**Housing stability**									
Unstable housing	21.6%	78.4%	0.714	32.0%	68.0%	0.001	27.8%	72.2%	0.002
Stable housing	14.8%	85.2%		21.7%	78.3%		18.4%	81.6%	
**Housing satisfaction**									
Unsatisfied housing	23.7%	76.3%	0.000	30.7%	69.3%	0.081	26.3%	73.7%	0.012
Satisfied housing	15.5%	84.5%		23.3%	76.7%		19.9%	80.1%	

**Table 3:** Regression results of housing status and stress.

Variables	Model 1	Model 2	Model 3	Model 4	Model 5
Housing tenure	−0.325 ** (0.116)				
Living space		−0.016 *** (0.004)			
Housing conditions			−0.066 (0.344)		
Housing stability				0.007 (0.078)	
Housing satisfaction					−0.405 *** (0.102)
Constant	3.131 *** (0.338)	3.124 *** (0.335)	2.991 *** (0.336)	2.982 *** (0.339)	2.875 *** (0.334)
*R* ^2^	0.021	0.028	0.013	0.013	0.029

Note: adjusted b coefficients are shown in the table; standard errors in parentheses; ** *p* < 0.01, *** *p* < 0.001; for visual clarity, the regression results of covariates (socio-demographic characteristics) are not shown in Table 3.

**Table 4:** Regression results of housing status and anxiety.

Variables	Model 6	Model 7	Model 8	Model 9	Model 10
Housing tenure	−0.122 (0.736)				
Living space		−0.066 * (0.027)			
Housing Conditions			1.698 (2.182)		
Housing stability				0.847 * (0.497)	
Housing satisfaction					−0.363 ** (0.079)
Constant	25.250 *** (2.153)	25.755 *** (2.134)	25.073 *** (2.133)	24.629 *** (2.149)	25.296 *** (2.135)
*R* ^2^	0.055	0.061	0.056	0.058	0.063

Note: adjusted b coefficients are shown in the table; standard errors in parentheses; * *p* < 0.05, ** *p* < 0.01, *** *p* < 0.001; for visual clarity, the regression results of covariates (socio-demographic characteristics) are not shown in Table 4.

**Table 5:** Regression results of housing status and depression.

Variables	Model 11	Model 12	Model 13	Model 14	Model 15
Housing tenure	0.205 (0.892)				
Living space		−0.058 * (0.033)			
Housing Conditions			3.242 (2.644)		
Housing stability				1.199 * (0.602)	
Housing satisfaction					−0.142 ** (0.027)
Constant	30.234 *** (2.611)	30.819 *** (2.591)	30.090 *** (2.585)	29.547 *** (2.604)	30.286 *** (2.589)
*R* ^2^	0.058	0.061	0.060	0.062	0.063

Note: adjusted b coefficients are shown in the table; standard errors in parentheses; * *p* < 0.05, ** *p* < 0.01, *** *p* < 0.001; for visual clarity, the regression results of covariates (socio-demographic characteristics) are not shown in Table 5.

**Table 6:** The moderating effects of subjective housing status on the relationship between objective housing status and stress.

Variables	Model 16	Model 17	Model 18	Model 19	Model 20
Housing tenure	−0.456 *** (0.119)	−0.507 *** (0.121)	−0.455 *** (0.119)	−0.453 *** (0.119)	−0.465 *** (0.118)
Living space	−0.016 *** (0.004)	−0.016 *** (0.004)	−0.016 *** (0.004)	−0.016 *** (0.004)	−0.016 *** (0.004)
Housing conditions	−0.074 (0.344)	−0.234 (0.354)	−0.075 (0.349)	0.110 (0.515)	−0.171 (0.348)
Housing stability	0.084 (0.079)	0.086 (0.078)	0.084 (0.079)	0.085 (0.079)	0.087 (0.079)
Housing satisfaction	−0.427 *** (0.103)	−0.371 ** (0.107)	−0.428 *** (0.109)	−0.431 *** (0.103)	−0.430 *** (0.103)
Housing tenure * Housing satisfaction		0.450 * (0.235)			
Living space * Housing satisfaction			0.000 (0.014)		
Housing conditions * Housing satisfaction				0.345 (0.717)	
Housing stability * Housing satisfaction					0.242 (0.233)
*R* ^2^	0.055	0.058	0.055	0.055	0.055

Note: * *p* < 0.05, ** *p* < 0.01, *** *p* < 0.001; for visual clarity, the regression results of covariates (socio-demographic characteristics) are not shown in Table 6.

**Table 7:** The moderating effects of subjective housing status on the relationship between objective housing status and anxiety.

Variables	Model 21	Model 22	Model 23	Model 24	Model 25
Housing tenure	−0.608 (0.764)	−1.257 (0.778)	−0.618 (0.766)	−0.638 (0.766)	−0.724 (0.760)
Living space	−0.073 ** (0.027)	−0.075 ** (0.027)	−0.074 ** (0.027)	−0.075 ** (0.027)	−0.076 ** (0.027)
Housing conditions	2.154 (2.220)	0.119 (2.270)	2.238 (2.250)	0.316 (3.318)	0.966 (2.231)
Housing stability	0.970 * (0.507)	0.999 * (0.503)	0.973 * (0.507)	0.966 * (0.507)	1.010 * (0.504)
Housing satisfaction	−0.362 ** (0.075)	−0.361 ** (0.072)	−0.360 ** (0.071)	−0.364 ** (0.081)	−0.363 ** (0.079)
Housing tenure * Housing satisfaction		1.416 (1.509)			
Living space * Housing satisfaction			−2.021 ** (0.092)		
Housing conditions * Housing satisfaction				3.446 (4.622)	
Housing stability * Housing satisfaction					5.372 *** (1.493)
*R* ^2^	0.066	0.066	0.076	0.067	0.078

Note: * *p* < 0.05, ** *p* < 0.01, *** *p* < 0.001; for visual clarity, the regression results of covariates (socio-demographic characteristics) are not shown in Table 7.

**Table 8:** The moderating effects of subjective housing status on the relationship between objective housing status and depression.

Variables	Model 26	Model 27	Model 28	Model 29	Model 30
Housing tenure	−0.472 (0.928)	−1.307 (0.944)	−0.467 (0.930)	−0.552 (0.928)	−0.595 (0.925)
Living space	−0.064 * (0.033)	−0.067 * (0.033)	−0.064 * (0.033)	−0.069 * (0.033)	−0.067 * (0.033)
Housing conditions	3.323 (2.695)	0.703 (2.753)	3.283 (2.732)	−1.661 (4.023)	2.065 (2.713)
Housing stability	1.265 * (0.615)	1.302 * (0.611)	1.263 * (0.616)	1.255 * (0.507)	1.307 * (0.613)
Housing satisfaction	−0.143 ** (0.027)	−0.139 ** (0.026)	−0.140 ** (0.026)	−0.142 ** (0.027)	−0.141 ** (0.026)
Housing tenure * Housing satisfaction		1.359 (1.830)			
Living space * Housing satisfaction			−3.010 ** (1.111)		
Housing conditions * Housing satisfaction				4.346 (5.605)	
Housing stability * Housing satisfaction					5.687 *** (1.815)
*R* ^2^	0.067	0.067	0.074	0.069	0.076

Note: * *p* < 0.05, ** *p* < 0.01, *** *p* < 0.001; for visual clarity, the regression results of covariates (socio-demographic characteristics) are not shown in Table 8.

## Data Availability

Data presented in this study are available on request from the corresponding author. Data are not publicly available due to data protection regulation.
